# Supplementary material for: Alternative polyadenylation diversifies post‐transcriptional regulation by selective RNA–protein interactions
Source: Mol Syst Biol. 2014 Feb 25;10(2):719. doi: 10.1002/msb.135068 (PMC4023391; doi:10.1002/msb.135068)
Supplement: Supplementary file 1 — Supplementary Figure 1 [file MSB-10-2-719-s01.pdf]

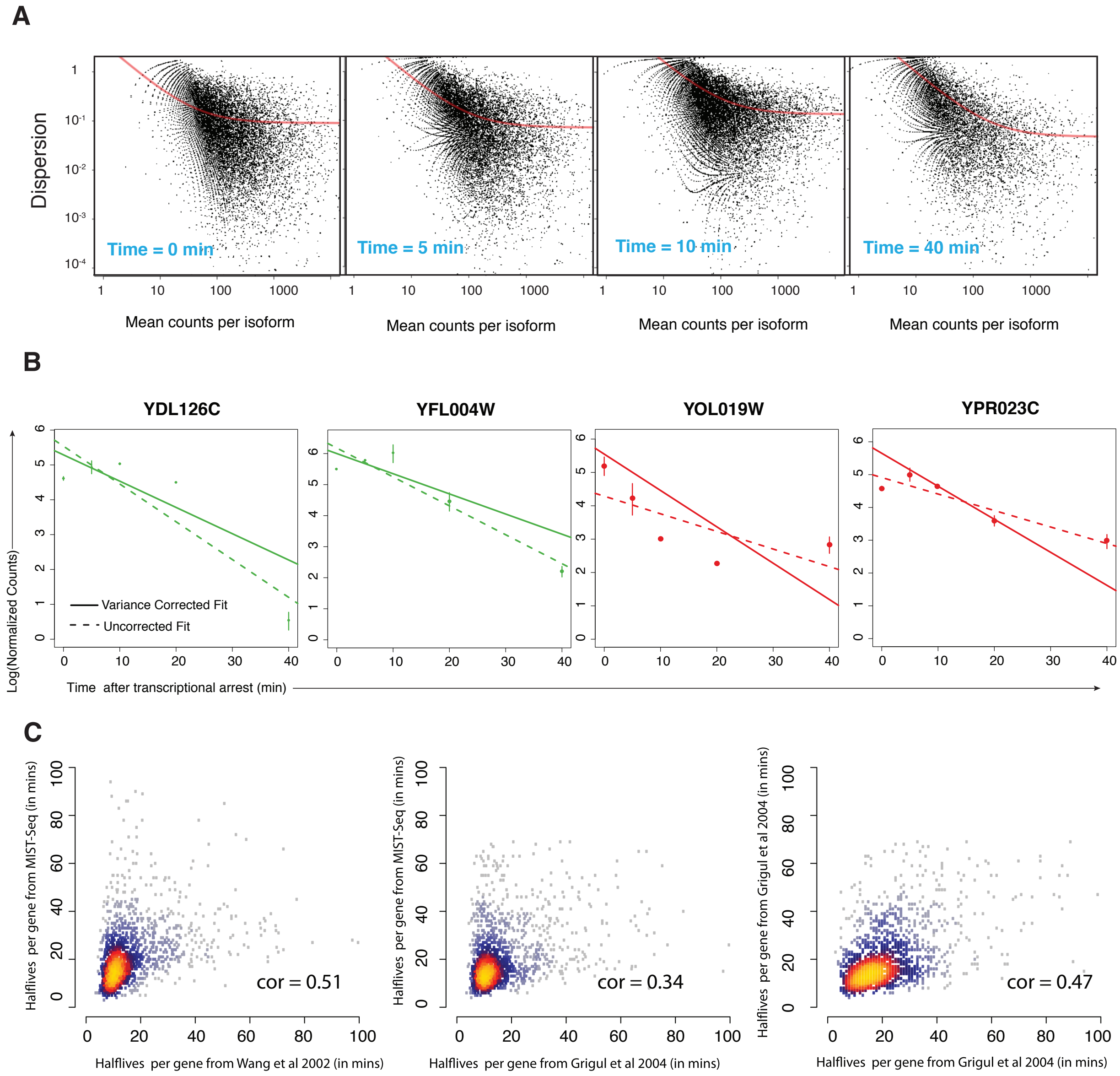

Figure S1. Motivation for using a weighted regression approach. (A) Plot of isoform dispersion (y-axis) versus mean counts (x-axis) between replicates across time points. The fitted estimates of regression (red line) show a shift to higher dispersion values at each time point. This indicates an increase of technical noise in the data due to lower counts at later time points. These fitted regressions were used to predict the dispersion for each count value (for each isoform at each time point), and subsequently used to weight the regression fit used to estimate the decay rates (Supplementary Experimental Procedures). (B) Examples where the unweighted regression leads to erroneous estimates of decay rates and consequently misidentification of isoforms with differential stability. (C) Correlation between cumulative (per gene) decay rates obtained from MIST-Seq compared to previous gene estimates of decay rates for about 2000 genes from previous studies (Grigull et al., 2004; Wang et al., 2002).
