# Supplementary material for: Alternative polyadenylation diversifies post‐transcriptional regulation by selective RNA–protein interactions
Source: Mol Syst Biol. 2014 Feb 25;10(2):719. doi: 10.1002/msb.135068 (PMC4023391; doi:10.1002/msb.135068)
Supplement: Supplementary file 2 — Supplementary Figure 2 [file MSB-10-2-719-s02.pdf]

A

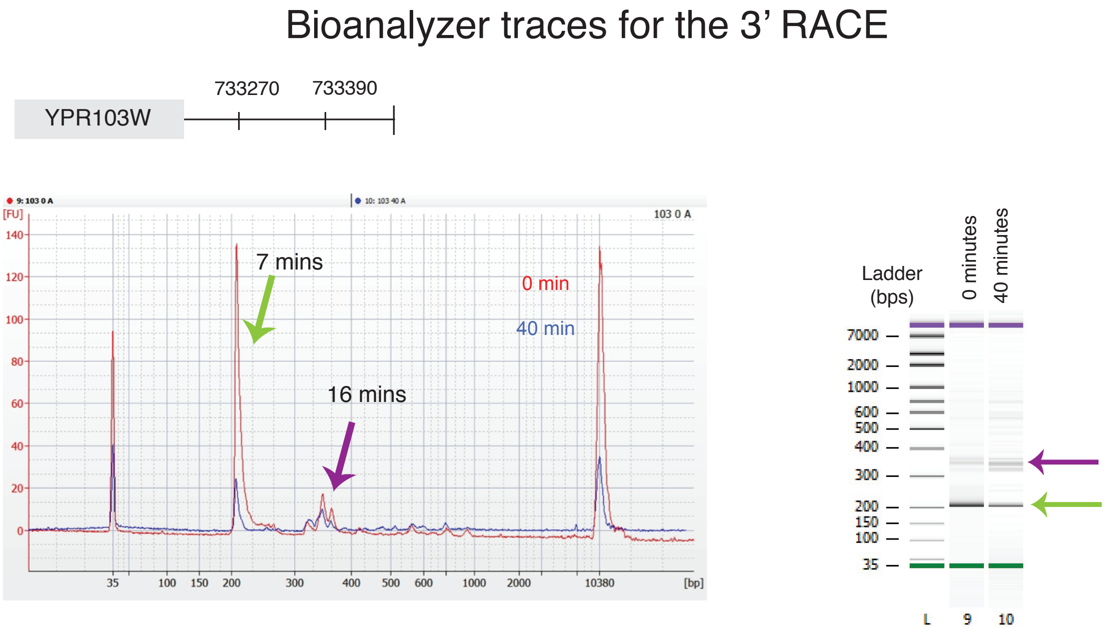

B

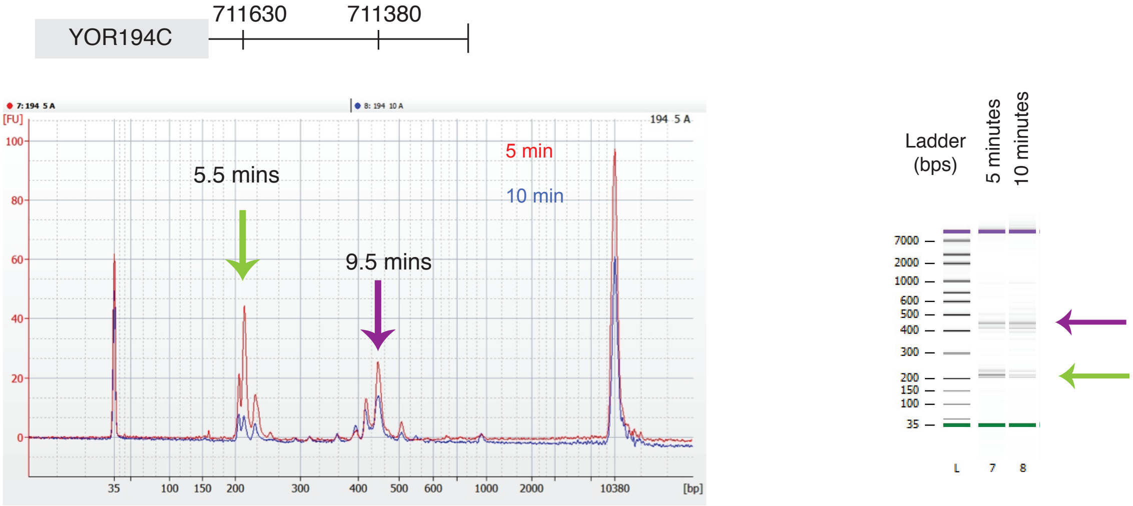

Figure S2. Confirmation of isoform-specific decay rate measurement by 3' RACE. To independently measure the relative change in abundance of stable and unstable polyadenylation isoforms after the transcriptional arrest, 3' RACE was performed for selected genes. Briefly RNA was reverse transcribed using an anchored oligo-dT primer including a common sequence. 3'UTRs were PCR amplified using a gene specific oligo and the common one inserted during reverse transcription. By resolving the amplified 3'UTRs in a capillary electrophoresis system we resolved small differences in the alternative 3'UTRs that would not be possible to resolve by Northern blot (where the small differences in 3'UTR will not produce big changes in the full-length RNA molecule and thus lead to an smear). (A) Bioanalyzer traces (left) and corresponding virtual Gel pictures (right) of two differentially decaying isoforms of gene YPR103W at time 0 minutes and 40 minutes after the transcriptional arrest. (B) Bioanalyzer traces (left) and corresponding Gel pictures (right) of two differentially decaying isoforms of gene

YOR194C at time 5 minutes and 10 minutes after the transcriptional arrest. In both the cases, the ratio of the abundance of the two isoforms changes in correspondence with the estimated difference in decay rates. Peaks at 35 and 10380 nt correspond to the internal Bioanalyzer standards (Agilent).
