# Supplementary material for: Alternative polyadenylation diversifies post‐transcriptional regulation by selective RNA–protein interactions
Source: Mol Syst Biol. 2014 Feb 25;10(2):719. doi: 10.1002/msb.135068 (PMC4023391; doi:10.1002/msb.135068)
Supplement: Supplementary file 3 — Supplementary Figure 3 [file MSB-10-2-719-s03.pdf]

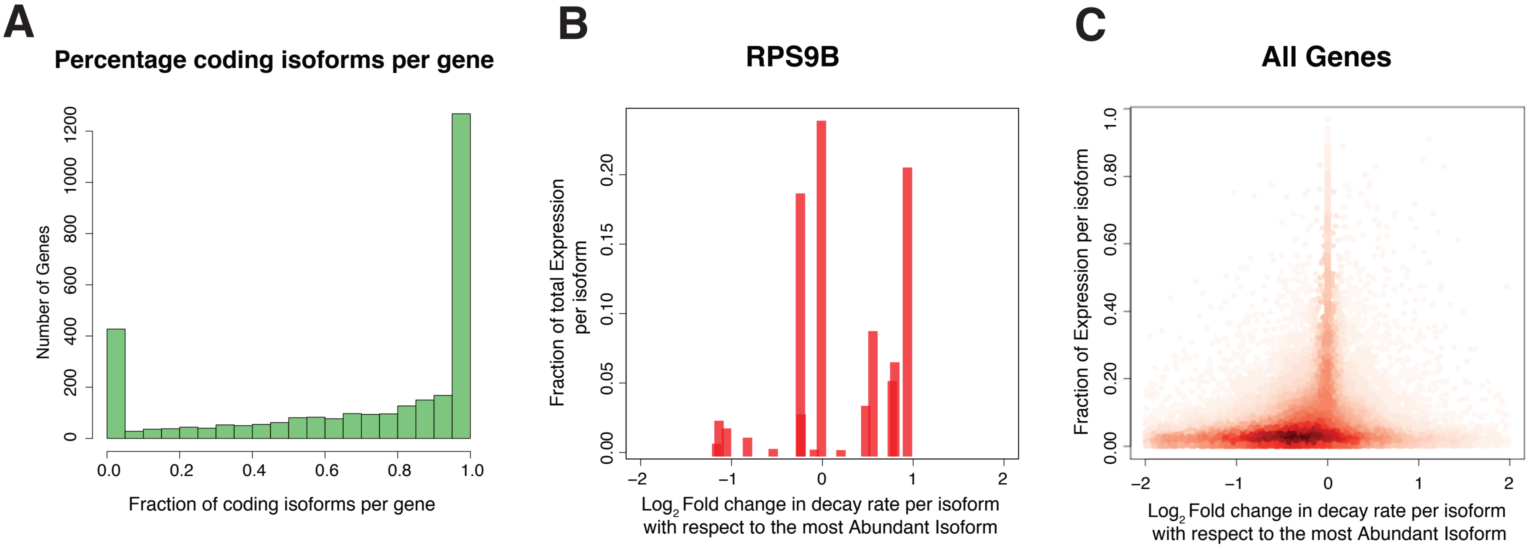

Figure S3. Relationship between isoform abundance and turnover within each gene. (A) Histogram of representing the fraction of coding isoforms in abundance contained per gene. (B) Plot between the fraction of total expression represented by an isoform versus the relative decay rate of the isoform with respect to the most abundant isoform for gene RPS9B (C) Previous plot made over all the genes measured with MIST-Seq
