# Supplementary material for: Alternative polyadenylation diversifies post‐transcriptional regulation by selective RNA–protein interactions
Source: Mol Syst Biol. 2014 Feb 25;10(2):719. doi: 10.1002/msb.135068 (PMC4023391; doi:10.1002/msb.135068)
Supplement: Supplementary file 4 — Supplementary Figure 4 [file MSB-10-2-719-s04.pdf]

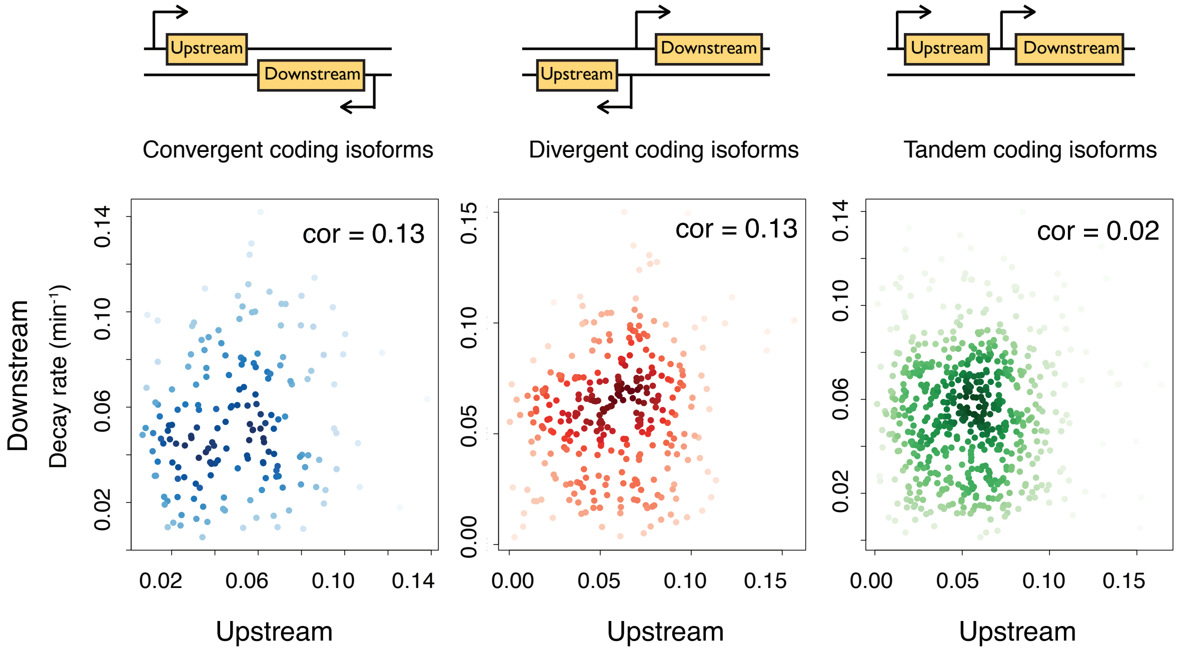

Figure S4. Decay rates of neighboring gene pairs do not show distinguishable patterns with respect to their orientation. Decay rates are shown for coding isoforms from 178 gene pairs in convergent orientation (blue), 409 gene pairs in divergent orientation (red), and 586 gene pairs in tandem orientation.
