# Supplementary material for: Alternative polyadenylation diversifies post‐transcriptional regulation by selective RNA–protein interactions
Source: Mol Syst Biol. 2014 Feb 25;10(2):719. doi: 10.1002/msb.135068 (PMC4023391; doi:10.1002/msb.135068)
Supplement: Supplementary file 5 — Supplementary Figure 5 [file MSB-10-2-719-s05.pdf]

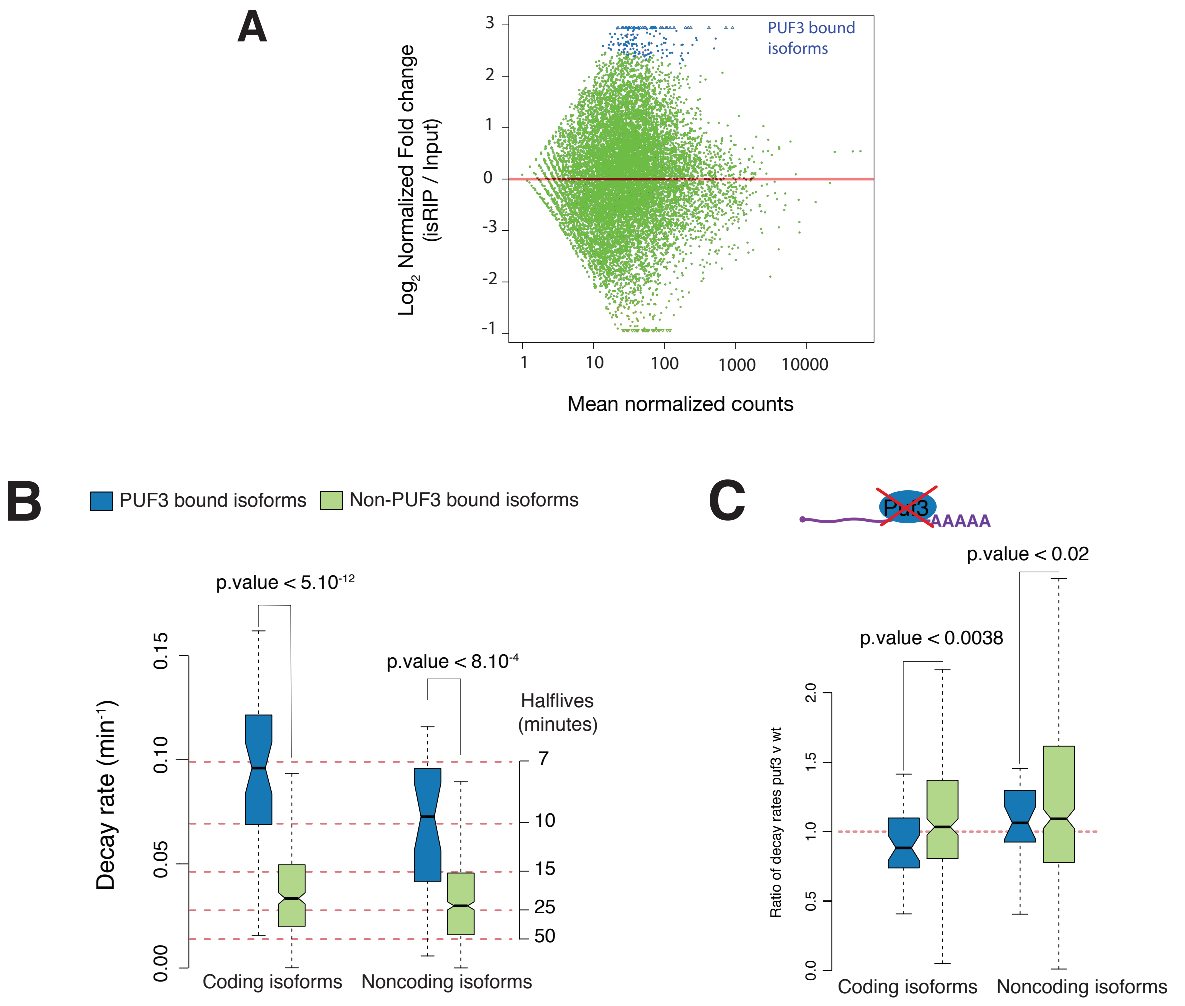

Figure S5. Determination of PUF3-bound isoforms and the changes in their stability. (A) Determination of significantly bound isoforms in isRIP. Plot of the per-isoform normalized fold change enrichment (y-axis) versus the mean normalized counts (x-axes) as calculated by DESeq2. Isoforms with fold change greater than 2 and statistically significant p-values (blue dots, 73 isoforms) were considered as being bound to PUF3. (B) Both coding (45) and noncoding (28) isoforms (left and right panels respectively) bound by PUF3 (blue) have lower stability in the wild-type strain than the corresponding isoforms for the same genes not bound by PUF3 (green). (C) Only PUF3 bound coding isoforms (left panel in blue) are significantly stabilized in a *puf3Δ* strain when comparing with the wild-type strain. (left panel).
