# Supplementary material for: Alternative polyadenylation diversifies post‐transcriptional regulation by selective RNA–protein interactions
Source: Mol Syst Biol. 2014 Feb 25;10(2):719. doi: 10.1002/msb.135068 (PMC4023391; doi:10.1002/msb.135068)
Supplement: Supplementary file 11 — Supplementary Methods [file MSB-10-2-719-s11.pdf]

Extended Experimental Procedures

Statistical methods for MIST-Seq analysis

Here we detail the statistical methods used to obtain robust estimates of decay rates for the 3’ isoforms of a gene and to calculate the significance in the difference between two decay rates. Here we apply it to *S. cerevisiae* but the same strategy could be extended to any time course measurement of transcript stabilities following transcriptional arrest.

Experimental outline

We profiled RNA abundance by 3’ specific isoform sequencing (3’T-fill) (Wilkening et al., 2013) at different time points following transcriptional arrest in *S. cerevisiae* strain *rpb1-1*. To normalize the sequencing counts of the decaying RNA population across each time point, we spiked in a small amount of foreign RNA (*i.e., S. pombe*) while preparing the libraries for sequencing. We used these "spiked-in" RNA as size factors to normalize the size of libraries at each time point when applying normalizing procedures. As a result, we see the number of reads mapping to *S. cerevisiae* decrease while the number of reads mapping to *S. pombe* (representing a constant number of molecules across time points) increase due to the decaying population of *S. cerevisiae* molecules.

Data processing

The details of our pipeline are provided in a Sweave document (Supplemental File S1) that allows others to reproduce our analysis and adapt the pipeline for their studies. We used our previously described sequencing pipeline (Wilkening et al., 2013) to obtain read counts for individual 3’ isoforms for each time point in our two biological replicate experiments. We use the *‘CountDataSet’* data structure of the "DESeq" Bioconductor package (Anders and Huber, 2010) to organize the data and later use the package's features to calculate dispersion and corresponding variance estimates. As a consequence of overall lower counts at the later time points, there are also more isoforms with lower count values. This increases the variance within the replicates and decreases the correlation between the replicates (Figure 2A). We therefore addressed these issues using the approach detailed below.

Decay rate calculation

In order to calculate the half-life for each isoform, we assume a single parameter exponential decay model

$$A(t) = A(0).e^{-ßt}$$

where ß denotes the decay rate, A(t) denotes normalized isoform expression counts at time point 't' after the transcriptional arrest, and A(0) denotes the normalized expression counts before transcriptional inhibition. Given this equation the half-life of the isoform can be calculated as *log*(2)/ß. The value of the decay rate ß is calculated from the slope of the linear regression on the above equation, log transformed:

$$log(A(t)) = log(A(0)) - ßt$$

Due to higher variance in the later time points, the regression fit might lead to inaccurate

estimations of decay rates by giving the later time points (with lower reproducibility due to lower counts) the same weight as the earlier time points (with higher reproducibility due to higher counts). We resolve this issue by assigning less weight to time points with lower counts. In general, this strategy amounts to assigning lesser weight to the isoform counts at the later time points following transcriptional arrest.

It is known from the weighted least squares theory that the optimal weight corresponds to 1/variance for each observation. If μ = E(k) denotes the expected value of the normalized count distribution its variance using the Negative Binomial model implemented in "DESeq" is given by

$$\mathrm{Var}(k) = \mu + \alpha\mu^2$$

where the parameter 'α' is commonly called the dispersion. For sufficiently smooth transformation f a first order Taylor approximation leads to the formula:

$$\mathrm{Var}[\ f(k)\ ] \approx (\ f'(E[k]\ )\ .\ \mathrm{Var}(k)$$

Applying the formula to our counts data represented by log-transformed values ln(k) for every isoform this becomes,

$$\mathrm{Var}[\ \ln(k)\ ] \approx 1/\mu + \alpha$$

In principle, this allows us to compute a variance for every observed count value. However, we do not have replication at the observation level. Thus, we use the replication on the gene level and estimate dispersion for every condition (time point) we have, using a robust fitting method implemented in DESeq. (Note that taking size factors into account changes the above formula slightly, the "shot-noise" is 1/raw counts instead of 1/normalized counts. Since the variance in our analysis is dominated by the dispersion this does not have a huge influence).
This allows us to forecast the variance of a single observation by the variance formula above, treating an observed count 'k' as an expected value. We then use these variance estimates to fit a weighted regression line to the log-transformed counts. Thus, the weight for a count is given by the reciprocal of:

$$1/k + \alpha$$

Comparing two decay rates
From any regression fit for each isoform we obtain a value for the slope (the decay rate) along with the standard error in estimating the slope. Therefore, to compare between two slopes we can derive the standard-normally distributed (assuming is true) test-statistic from the central limit theorem:

$$(\beta_1 - \beta_2)/\sqrt{}\ se(\beta_1)^2 + se(\beta_2)^2$$

where β_1, β_2 are the estimated decay rates for 2 isoforms and se(β_1), (se(β_2) are the standard errors in estimating these decay rates.
