# Supplementary material for: Alternative polyadenylation diversifies post‐transcriptional regulation by selective RNA–protein interactions
Source: Mol Syst Biol. 2014 Feb 25;10(2):719. doi: 10.1002/msb.135068 (PMC4023391; doi:10.1002/msb.135068)
Supplement: Supplementary file 12 — Supplementary Legends [file MSB-10-2-719-s12.pdf]

Supplementary Table S1. Decay rates of isoforms associated with specific Gene Ontology terms.

Supplementary Table S2. Results from ANOVA followed pair-wise t-tests on the decay rates of isoforms belonging to each of the eight transcript isoform categories in Figure 4 .

Supplementary Table S3. Isoform pairs with significantly different decay rates in wild-type yeast.

Supplementary Table S4. Stabilities for isoforms with and without RBP motif for genes with RBP motif.

Supplementary Table S5. Stabilities of PUF3-bound isoforms in wild-type yeast and their relative stabilities in *puf3Δ.*

Supplemental File S1. Sweave document with a detailed computational top-down analysis pipeline that can be used to reproduce our MIST-Seq analysis and easily adapted for future studies.
