# Supplementary material for: Alternative polyadenylation diversifies post‐transcriptional regulation by selective RNA–protein interactions
Source: Mol Syst Biol. 2014 Feb 25;10(2):719. doi: 10.1002/msb.135068 (PMC4023391; doi:10.1002/msb.135068)
Supplement: Supplementary file 13 — Sweave document [file MSB-10-2-719-s13.zip › MIST-Seq_v03.pdf]

MIST-Seq Pipeline

Ishaan Gupta, Bernd Klaus

European Molecular Biology Laboratory (EMBL),
Heidelberg, Germany

gupta@embl.de

January 20, 2014

1 Introduction

Heterogeneity in the length of transcribed molecules of the same gene has been uncovered by our group and others [?]. This difference in length of the transcripts is of relevance with respect to the 3' ends of genes as this region of genes is enriched for regulatory sequences like RNA binding protein (RBP) sites or miRNA sites and are evolutionary conserved [?]. Therefore, two transcript molecules of the same gene (or 3' isoforms), owing to their different 3'UTRs , can encode for different RBP sites. A direct consequence of this differential RBP sites is on halflives of the isoforms.

We demonstrate for the first time on a genome-wide scale, that about 10 p.c. of yeast genes have 1 or more 3' isoforms of the same gene with different halflives. We further demonstrate that this difference in haflives is affected by the presence of RBP motifs (as taken from [?]). Consequently, we discover a RBP-code whereby RBPs might interact with each other and selectively regulate different isoforms of the same gene. This is relevant in the context of yeast cells as majority of transcripts are present at 1 molecule/cell implying that every cell might have a different isoform of the same gene. This result significantly contributes to explaining variegated gene expression in cells and the origins of transcription noise in homogeneous cell populations.

In this document, we entail the pipeline and assumptions used to arrive at the conclusions detailed earlier. We use the modified technique of 3' Isoform profiling ([?]) to obtain quantitative measure of a decaying population 3'isoforms in BY4741-rpb11 budding yeast strain grown in rich media after transcriptional arrest. The first section deals with the nature of data and experimental controls used. The second section details the problems and potential solutions with calculations of half-life parameter based on the expression count data. The third section deals with the results obtained.

2 Data acquisition

We used a yeast strain with the BY background containing a rpb1-1 allele of the largest subunit of RNA Polymerase I which is inactivated when grown in 37°C medium. We grew 2 independent cultures of the strain to a density of approximately 2 million cells/ml (log phase) in 24 °C which is permissive for its growth such that it attained a steady state RNA population. Then the culture was shifted to 37 °C inactivating further RNA transcription and resulting in only RNA decay. In order to quantify this decay we collected cells and extracted RNA from them after the shift at 5 mins, 10 mins, 20 mins and 40 mins and subjected the RNA to 3' Tag-sequencing. In order to normalize the counts each time point due to decaying RNA population we spiked in a small amount of S.pombe RNA while preparing the libraries for sequencing. We later use this "spiked-in" pombe RNA as size factors to normalize the size of libraries at each time point when applying normalizing procedures. Our data consists of counts.

```
[1] 1
[1] 2
[1] 3
[1] 4
[1] 5
[1] 6
[1] 7
[1] 8
[1] 9
[1] 10
```

```
 chr [1:1172660] "chr01.-.3513" "chr01.-.5011" "chr01.-.5029" ...
```

```
 chr [1:374931] "chr01.-.3513" "chr01.-.5011" "chr01.-.5029" ...
```

```
'data.frame':        101098 obs. of  11 variables:
 $ space  : Factor w/ 374931 levels "chr01.-.100017",..: 3344 3670 3042 3120 3123 3142 3183 3184
 $ rep1t0 : int  1 2 5 12 4 2 2 6 5 2 ...
 $ rep1t5 : int  NA NA NA 10 1 NA NA NA 1 2 ...
 $ rep1t10: int  NA NA NA 2 NA 20 NA NA NA NA ...
 $ rep1t20: int  1 NA NA 6 4 12 NA NA NA NA ...
 $ rep1t40: int  2 NA NA NA 1 NA NA NA NA NA ...
 $ rep2t0 : int  12 1 1 10 10 2 5 7 1 1 ...
 $ rep2t5 : int  2 NA NA 2 5 NA NA NA NA NA ...
 $ rep2t10: int  3 NA 2 7 5 1 NA NA NA NA ...
 $ rep2t20: int  2 NA 2 4 NA 1 NA NA NA NA ...
 $ rep2t40: int  NA NA NA NA NA NA NA NA NA NA ...
```

```
'data.frame':        78434 obs. of  11 variables:
 $ space  : Factor w/ 374931 levels "chr01.-.100017",..: 3120 3123 3216 3218 3233 3236 3239 3260
 $ rep1t0 : int  12 4 5 2 12 5 1 11 11 31 ...
 $ rep1t5 : int  10 1 1 2 4 6 1 2 3 17 ...
 $ rep1t10: int  2 NA NA NA NA 20 NA 21 NA 28 ...
 $ rep1t20: int  6 4 NA NA 8 NA NA NA 10 16 ...
 $ rep1t40: int  NA 1 NA NA 1 NA NA 10 NA 21 ...
 $ rep2t0 : int  10 10 1 1 3 13 3 16 5 8 ...
 $ rep2t5 : int  2 5 NA NA 4 4 3 17 NA 16 ...
```

```
 $ rep2t10: int  7 5 NA NA 3 5 1 4 3 34 ...
 $ rep2t20: int  4 NA NA NA 5 5 NA 7 1 25 ...
 $ rep2t40: int  NA NA NA NA 4 NA 1 6 1 5 ...
```

We use the CountDataSet sructure of the "DESeq" [?] package to organize the data and later use the package's features to calculate dispersion and corresponding variance estimates. First, we calculate the size factors. They correspond to the total number of counts for the pombe spike-in (scaled to be close to 1). We have two replicates per time point, denoted by B and C and four time points in total: 0, 5, 10, and 40 minutes.

```
> options(width=20)
> # library sizes which can be later used as DESeq size factors
>
> tmp.sizes = sapply( 1:10, function(x) sum(pombe[[x]]$count) )
> tmp.sizes = tmp.sizes/30000
> Ishaan.data.cds = newCountDataSet(data12[,-1], conditions = rep(c("t0", "t5", "t10","t20", "t40
> sampleNames(Ishaan.data.cds)
```

```
 [1] "rep1t0"
 [2] "rep1t5"
 [3] "rep1t10"
 [4] "rep1t20"
 [5] "rep1t40"
 [6] "rep2t0"
 [7] "rep2t5"
 [8] "rep2t10"
 [9] "rep2t20"
[10] "rep2t40"
```

```
> conditions(Ishaan.data.cds)
```

```
 rep1t0  rep1t5
     t0      t5
rep1t10 rep1t20
    t10     t20
rep1t40  rep2t0
    t40      t0
 rep2t5 rep2t10
     t5     t10
rep2t20 rep2t40
    t20     t40
5 Levels: t0 ...
```

```
> sizeFactors(Ishaan.data.cds) = tmp.sizes
>
```

The sample names for the CountDataSet correspond to the replicate names (B and C) combined with the time points, which represent the conditions. Thus, for every condition (= time point) we have two replicates.

```
> options(width=40)
> sampleNames(Ishaan.data.cds)
```

```
 [1] "rep1t0"  "rep1t5"  "rep1t10"
 [4] "rep1t20" "rep1t40" "rep2t0"
 [7] "rep2t5"  "rep2t10" "rep2t20"
[10] "rep2t40"
```

```
> conditions(Ishaan.data.cds)
```

```
 rep1t0  rep1t5 rep1t10 rep1t20 rep1t40
     t0      t5     t10     t20     t40
 rep2t0  rep2t5 rep2t10 rep2t20 rep2t40
     t0      t5     t10     t20     t40
Levels: t0 t10 t20 t40 t5
```

```
> # size factors as calculated for the common pombe "spike-in"
> sizeFactors(Ishaan.data.cds)
```

```
   rep1t0    rep1t5   rep1t10   rep1t20
0.8372667 1.0756000 1.0242667 1.7676333
  rep1t40    rep2t0    rep2t5   rep2t10
2.3085667 0.8382333 0.7765333 1.1675667
  rep2t20   rep2t40
1.2800667 1.6117667
```

3 Normalization and Half-life calculation

As expected after transcriptional inhibition we see that the total number of raw as well as normalized counts decreases.

```
> options(width=20)
> ## raw counts
> apply(counts(Ishaan.data.cds, normalized = FALSE), MARGIN = 2, sum)
```

```
 rep1t0  rep1t5
6904147 6689259
rep1t10 rep1t20
7637025 6815208
rep1t40  rep2t0
5315785 5539240
 rep2t5 rep2t10
5209356 5794945
rep2t20 rep2t40
4956961 3473359
```

```
> ## normalized counts
> apply(counts(Ishaan.data.cds, normalized = TRUE), MARGIN = 2, sum)
```

```
 rep1t0  rep1t5
8246055 6219095
rep1t10 rep1t20
7456091 3855555
```

```
rep1t40  rep2t0
2302634 6608232
 rep2t5 rep2t10
6708477 4963267
rep2t20 rep2t40
3872424 2155001
```

As a consequence of a lower overall counts at the later time points there are also more single isoforms with lower count values. This increases the variance within the replicates and furthermore decreases the correlation between the replicates increases as shown in the heat–scatterplot.

```
null device
          1
```

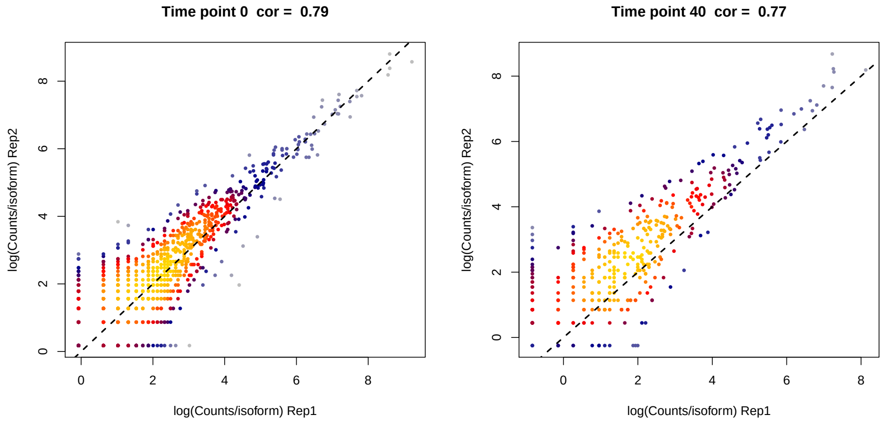

Figure 1: Two scatterplots of log of counts per isoform for time point 0 mins (before transcriptional inhibition) and the latest time point (40 mins) after inhibition. The later we collect the RNA after inhibition the variance we see in the data due to lower counts as a result of decayed RNA molecules.

In order to calculate the halflives, we assume a single parameter exponential decay model

$$\mathrm{A(t)} = A(0) * exp(-\eta * t)\,,$$

where η denotes the decay rate, A(t) denotes normalized isoform expression counts at time 't' and A(0) denotes the normalized expression counts before transcriptional inhibition. Given this equation the half-life of the isoform can be calculated as $-\ln(2)/\eta$. Therefore, the value of η can be calculated from the slope of the linear regression on the log-transformed above equation , $\ln(A(t)) = \ln(A(0)) - \eta *$.

Hence, due to higher variance in the later time points the regression fit might lead to improper estimation of line slopes and thus the half-lives . In order words, if we were to fit a regression–line to the $\log_2$-transformed values at each time-point, we would need to give less weights to

observations corresponding to latter time points due to higher variances. It is known from weighted least squares theory that the optimal weight corresponds to 1/var for each observation.

If $\mu = E(K)$ denotes the expected value of the normalized count distribution K its variance using the Negative Binomial model implemented in DESeq is given by

$$\mathrm{Var(K)} = \mu + \alpha\mu^2\,,$$

where the parameter alpha is commonly called dispersion. For any sufficiently smooth transformation f a simple first order Taylor approximation leads to the formula formula

$$\mathrm{Var}[f(K)] \approx \Big(f^{'}(E[K])\Big)^2 \cdot \mathrm{Var}[K]$$

applied to $f = \ln$ this becomes

$$\begin{aligned}\mathrm{Var}[\log_2(K)] = \mathrm{Var}\left[\ln(K)\right] = \mathrm{Var}[\ln(K)] \\ \approx \frac{1}{\mu^2}(\mu + \alpha\mu^2) = \frac{1}{\mu} + \alpha\end{aligned}$$

In principle, this allows us to compute a variance for every observed count value. However we do not have replication on the observation level. Thus, we use the replication on the gene level and estimate a dispersion for every condition (time point) we have, using a robust fitting method implemented in DESeq.

```
> options(width=40)
> ### estimate dispersions per condition
> Ishaan.data.cds = estimateDispersions( Ishaan.data.cds, method="per-condition",
+  sharingMode="fit-only")
```

This gives us the possibility of forecasting the variance of a single observation by the variance formula above, treating an observed count k as an expected value. We then use these variance estimates to fit a weighted regression line to the ln transformed counts. Thus, the weight for a count k is given by the reciprocal of: $\frac{1}{k} + \alpha$.

Note that this appraoch is also follwed in the "Voom" function of the limma package and seems to be very reasonable for modeling the mean–variance relationship in RNA–Seq data [?].

The following code shows the computation of the condition specific dispersion estimation.

```
> #### get dispersion functions
> #### they take normalized mean expression value as input and give the corresponding
> #### dispersion estimate
>
> for( k in 1:5){
+ eval(parse(text = paste("dispFunc", levels(conditions(Ishaan.data.cds))[k] ," <- ",
+ "fitInfo( Ishaan.data.cds,
+ name= levels(conditions(Ishaan.data.cds))[k])$dispFunc" , sep = "") ))
+
+ }
> data.cds = Ishaan.data.cds
> variances.log <- data.frame( matrix(NA, nrow = dim(data.cds)[1], ncol = dim(data.cds)[2],
+ dimnames = list(featureNames(data.cds), sampleNames(data.cds)) ))
> idx = vector("list",5)
```

```
> disp = vector("list",5)
> timePoints = c( "t0","t5","t10","t20","t40")
> dispFunct = c(dispFunct0,   dispFunct5,        dispFunct10,  dispFunct20,  dispFunct40)
> for( k in 1:5) {
+ ### obtain dispersions for timePoint indexed at 'k' position in 'timePoints' vector
+ idx[[k]] = conditions(data.cds) == timePoints[k]
+ disp[[k]] = apply(counts(data.cds, normalized=F)[, idx[[k]]], MARGIN = 2, dispFunct[[k]] )
+
+ ### obtain variances for timePoint indexed at 'k' position in 'timePoints' vector
+ variances.log[, idx[[k]] ] = (1/counts(data.cds, normalized=F)[, idx[[k]] ] + disp[[k]] )
+ print(head(variances.log[, idx[[k]] ]))
+
+ }

     rep1t0    rep2t0
21 0.479310 0.5559789
23 1.245999 0.5559789
30 1.015992 4.6960997
31 2.396033 4.6960997
33 0.479310 1.6293436
34 1.015992 0.4498220
      rep1t5   rep2t5
21 0.8511848 3.976158
23 7.8823753 1.632428
30 7.8823753      Inf
31 3.9761584      Inf
33 2.0230499 2.023050
34 1.3720137 2.023050
     rep1t10  rep2t10
21 7.7830471 2.319677
23       Inf 3.193816
30       Inf      Inf
31       Inf      Inf
33       Inf 5.233474
34 0.8992004 3.193816
    rep1t20  rep2t20
21 1.481310 2.210920
23 2.210920      Inf
30      Inf      Inf
31      Inf      Inf
33 1.116506 1.773154
34      Inf 1.773154
    rep1t40  rep2t40
21      Inf      Inf
23 9.035981      Inf
30      Inf      Inf
31      Inf      Inf
33 9.035981 2.291058
34      Inf      Inf
```

```
> head(variances.log)

     rep1t0    rep1t5   rep1t10
21 0.479310 0.8511848 7.7830471
23 1.245999 7.8823753       Inf
30 1.015992 7.8823753       Inf
31 2.396033 3.9761584       Inf
33 0.479310 2.0230499       Inf
34 1.015992 1.3720137 0.8992004
    rep1t20  rep1t40    rep2t0   rep2t5
21 1.481310      Inf 0.5559789 3.976158
23 2.210920 9.035981 0.5559789 1.632428
30      Inf      Inf 4.6960997      Inf
31      Inf      Inf 4.6960997      Inf
33 1.116506 9.035981 1.6293436 2.023050
34      Inf      Inf 0.4498220 2.023050
    rep2t10  rep2t20  rep2t40
21 2.319677 2.210920      Inf
23 3.193816      Inf      Inf
30      Inf      Inf      Inf
31      Inf      Inf      Inf
33 5.233474 1.773154 2.291058
34 3.193816 1.773154      Inf
```

We illustrate the the difference between a weighted fit and an ordinary fit using isoform 25 in line 4 of the data. As you can see, the line obtained by the weighted fit is closer to the higher, more reliable count values which have less variance.

```
> options(width=40)
> ### compute example linear model and corresponding halflife for isoform 23 (line No. 4)
> isoId=234
> X <- rep(c(0,5,10,20,40) ,2 )
> Y <- log(counts(Ishaan.data.cds, normalized=T)[ isoId, ])
> ### fit model without weights
> lm.no.weights <- lm(Y ~ X)
> summary(lm.no.weights)

Call:
lm(formula = Y ~ X)

Residuals:
     Min       1Q   Median       3Q
-0.74353 -0.10477 -0.01572  0.20441
     Max
 0.52023

Coefficients:
             Estimate Std. Error
(Intercept)  6.321572   0.163393
X           -0.089537   0.007926
```

```
            t value Pr(>|t|)
(Intercept)   38.69 2.19e-10 ***
X            -11.30 3.39e-06 ***
---
Signif. codes:  0 '***' 0.001 '**' 0.01 '*' 0.05 '.' 0.1 ' ' 1

Residual standard error: 0.3544 on 8 degrees of freedom
Multiple R-squared:  0.941,       Adjusted R-squared:  0.9336
F-statistic: 127.6 on 1 and 8 DF,  p-value: 3.392e-06
```

```
> ### Weights are equal to 1/variances
> W <- 1/as.vector(variances.log[isoId, ], mode = "numeric")
> lm.weights <- lm(Y ~ X, weights = W)
```

From such a regression fit we obtain a value for the slope (the decay rate) for each isoform along with it's standard error estimate. Therefore, to compare between two slopes we can derive the standard–normally distributed (assuming H_0 is true) test–statistic from the central limit theorem:

$$\frac{\text{slope}_1 - \text{slope}_2}{\sqrt{\text{SE}_1^2 + \text{SE}_2^2}}$$

```
null device
          1
```

We can now test for different slopes (= decay rates) between different isoforms using a two–sided z–test:

```
> options(width=40)
> # Function checkSlope returns the p-value after performing the z-test on 2 slope values
> checkSlopes = function(s1,sd1,s2,sd2){
+ eta = (s1-s2)/sqrt(sd1^2+sd2^2)
+ pval = 2-2*pnorm(abs(eta))
+ pval
+ }
```

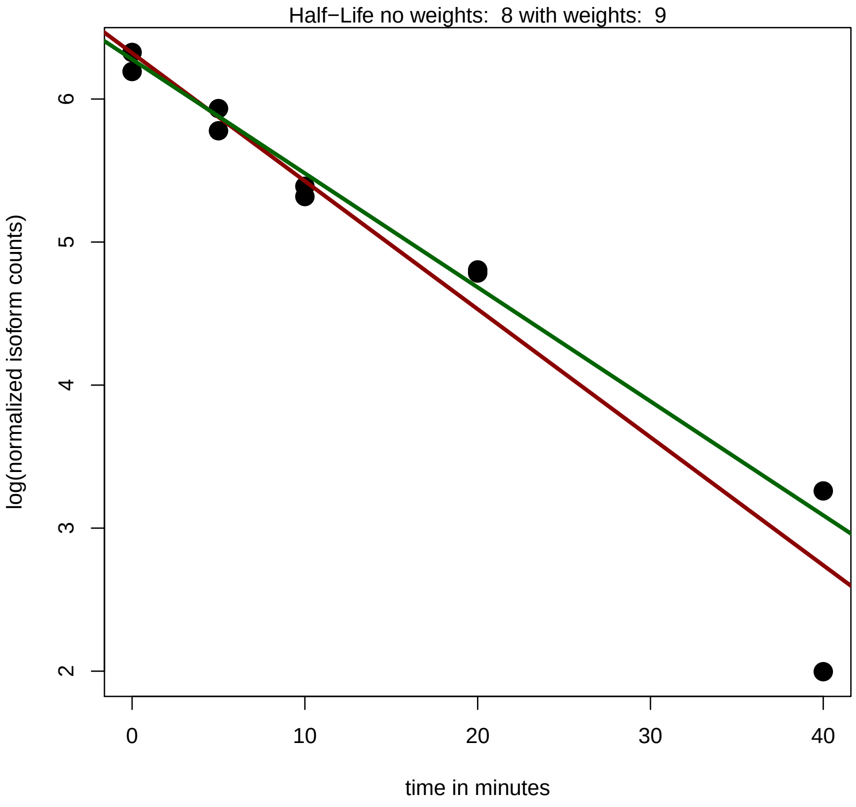

Figure 2: Comparison of non-weighted(red) vs weighted fit(green). The non-weighted is heavily influenced by the latter "unreliable" time point value as a result the halflife estimate is possibly erroneous.

4 Results

Therefore, we employ the strategy employed above to calculate the halflives for all the isoforms.

```
> options(width=40)
> X = rep( c(0,5,10,20,40),2 )
> Y <- log(counts(Ishaan.data.cds, normalized=T) )
> Z <- vector("list", nrow(Y))
> for( i in 1:nrow(Y) ){
+ W <- 1/as.vector(variances.log[i, ], mode = "numeric")
+ Z[[i]] = lm(Y[i,]~X,weights=W)
+ #if( i%% 1000 == 0 ) print(i)
+ }
> test = t(sapply( Z, function(x) summary(x)$coefficients[2,1:2] ))
> test = data.frame(test)
> test$space = data12$space
>
```

We now, move to proving our first result i.e. there are about 10 p.c. yeast genes with 1 or more isoforms with different halflives. We use the IRanges package for this.

```
> options(width=20)
> # Get the indices of isoforms overlapping the annotated gene and its 3' UTR
> isoforms_gene = overlapList(Ranges_isoforms,Ranges_full)
> # Constructing the dataframes with isoforms and genes
> df_isoforms = getRangesDataframe(Ranges_isoforms)
> df_genes = getRangesDataframe(Ranges_full)
> df_genes$name = Ranges_full@values@unlistData@listData[[1]]
> df_isoforms$slope = Ranges_isoforms@values@unlistData@listData[[1]]
> df_isoforms$se = Ranges_isoforms@values@unlistData@listData[[2]]
> pvalTests = vector("list",7272)
> for( i in 1:7272){
+
+ if(length(isoforms_gene[[i]]) >1) {
+     mix=combn2(isoforms_gene[[i]])
+
+     vals = sapply( 1:nrow(mix) ,
+     function(x)
+     checkSlopes(df_isoforms$slope[mix[x,1]],df_isoforms$se[mix[x,1]],df_isoforms$slope[mix[x,2]
+     )
+
+     pvalTests[[i]] = data.frame(mix)
+     pvalTests[[i]]$pval = p.adjust(vals,method="BH")
+     }
+ #if(i %% 1000 ==0) print(i)
+ }
> hits0  = which(sapply( pvalTests, function(x) sum(x$pval < 0.1) )  > 0 )
> str(hits0)

 int [1:1190] 14 16 22 25 26 33 36 38 43 57 ...
```

Example for genes with isoforms of different stabilities

```
> # Gene at index 4 has two isoforms with variedly different slopes and hence isoform stabilities
> df_genes[ hits0[4],]

           start
chr01.-.25 87498
             end
chr01.-.25 92514
           width
chr01.-.25  5017
              name
chr01.-.25 YAL029C

> hits[[4]]

           start
chr01.-.42 87691
chr01.-.43 87692
chr01.-.47 87743
chr01.-.52 87767
chr01.-.55 87787
             end
chr01.-.42 87691
chr01.-.43 87692
chr01.-.47 87743
chr01.-.52 87767
chr01.-.55 87787
           width
chr01.-.42     1
chr01.-.43     1
chr01.-.47     1
chr01.-.52     1
chr01.-.55     1
                  slope
chr01.-.42 -0.051171581
chr01.-.43 -0.008186356
chr01.-.47 -0.049310402
chr01.-.52 -0.050181583
chr01.-.55 -0.057220738
                    se
chr01.-.42 0.004788553
chr01.-.43 0.010729927
chr01.-.47 0.009151774
chr01.-.52 0.003751964
chr01.-.55 0.008596414

null device
          1
```

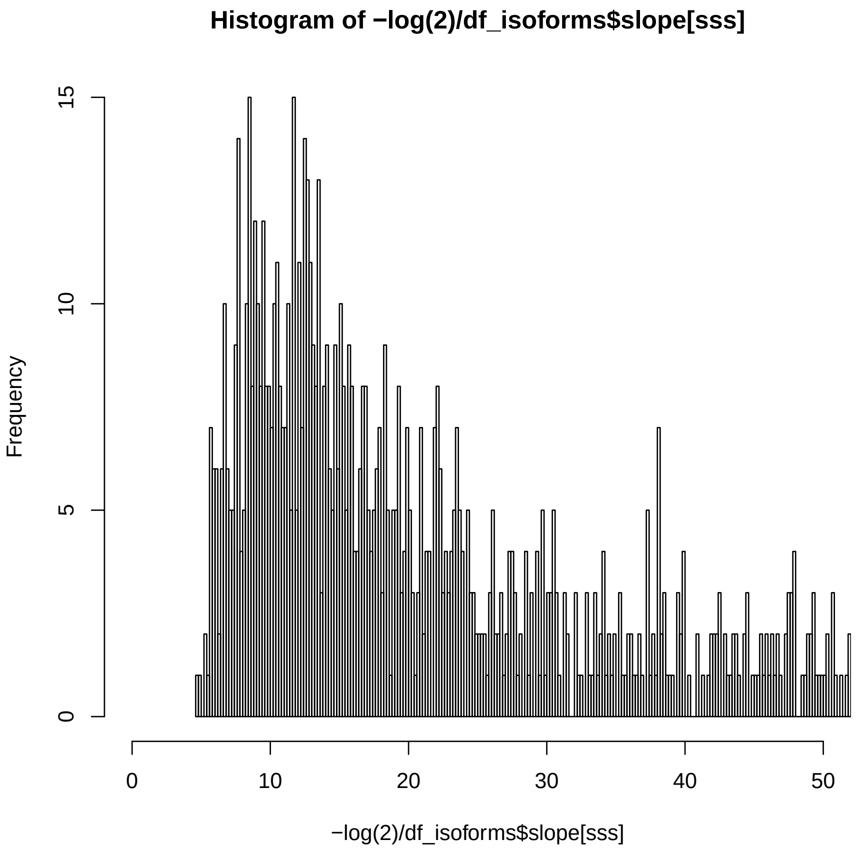

Figure 3: Histogram of 1000 randomly chosen isoforms. These distributions have 11 mins median halflives.

```
null device
          1
```

When we divide the isoforms in 2 groups, isoforms with a RBP site and isoforms without the RBP site for all those genes the particular RBP site. We find that most of the ”PUF” proteins are destabilizing.

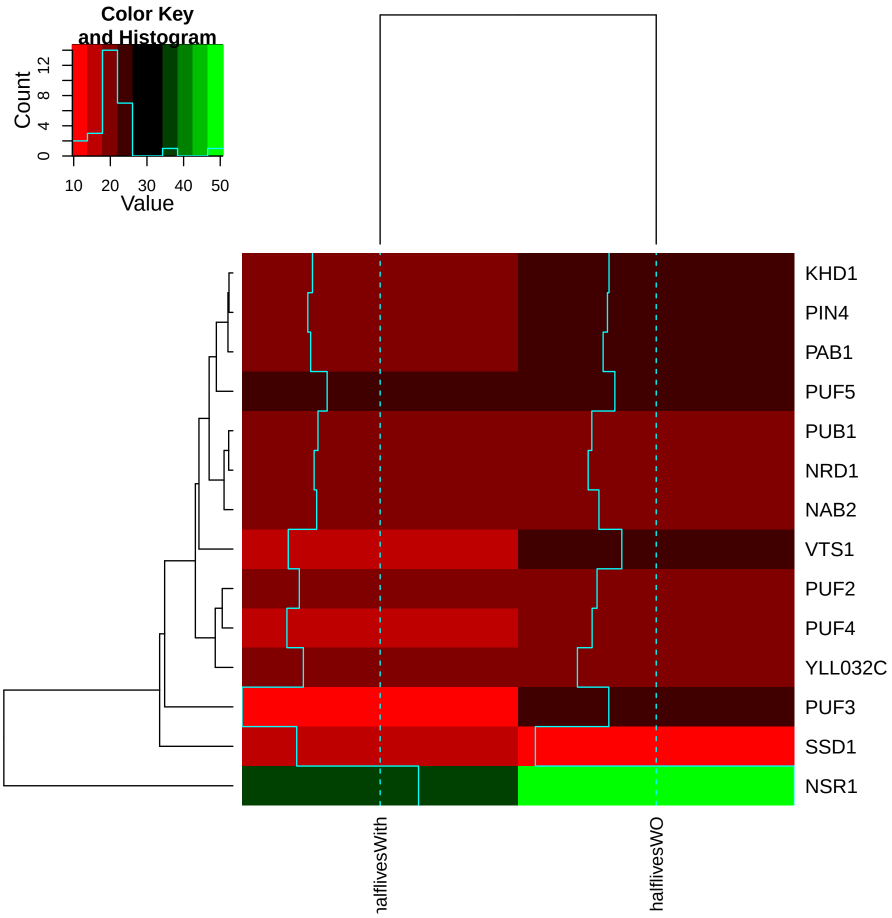

Figure 4: RBP-wise stability of isoforms. All the PUFproteins are destabilizing as is PAB1 which is known to be involved in recruiting the complexes which lead to RNA degradation

References

[Anders and Huber(2010)] Simon Anders and Wolfgang Huber. Differential expression analysis for sequence count data. *Genome Biology*, 11:R106, 2010. doi: 10.1186/gb-2010-11-10-r106. URL `http://genomebiology.com/2010/11/10/R106/`.

[Law et al.(2013)] CW Law, Y Chen, W Shi, and GK Smyth. Voom! precision weights unlock linear model analysis tools for rna-seq read counts. *Preprint - Bioinformatics Division, Walter and Eliza Hall Institute of Medical Research, Melbourne, Australia*, 2013.

[Pelechano (2010)] Vicente Pelechano, Wu Wei, Lars Steinmetz Extensive transcriptional heterogeneity revealed by isoform profiling. *Nature 2013 May 2;497(7447):127-31*, doi: 10.1038/nature12121. URL http://www.nature.com/nature/journal/v497/n7447/full/nature12121.html.

[Wilkening (2013)] Wilkening, S., Pelechano, V., Jarvelin, A.I., Tekkedil, M.M., Anders, S., Benes, V. Steinmetz, L.M. An efficient method for genome-wide polyadenylation site mapping and RNA quantification *Nucleic Acids Res. 2013 Mar 1;41(5):e65* doi: 10.1093/nar/gks1249. URL http://europepmc.org/articles/PMC3695521/.

[Hogan (2008)] Hogan, D. J. and Riordan, D. P. and Gerber, A. P. and Herschlag, D. and Brown, P. O. Diverse RNA-binding proteins interact with functionally related sets of RNAs, suggesting an extensive regulatory system *PLoS Biol.2008, 6, 10:e255* doi: 10.1371/journal.pbio.0060255. URL http://www.plosbiology.org/article/info%3Adoi%2F10.1371%2Fjournal.pbio.0060255.

[Freeberg (2013)] Freeberg, MA, Han, T, Moresco, JJ, Kong, A, Yang, YC, Lu, ZJ, Yates, JR, Kim, JK Pervasive and dynamic protein binding sites of the mRNA transcriptome in Saccharomyces cerevisiae *Genome Biol. 2013, 14, 2:R13* URL http://genomebiology.com/content/14/2/R13
